# Effects of *Lactobacillus paracasei* CNCM I-4034, *Bifidobacterium breve* CNCM I-4035 and *Lactobacillus rhamnosus* CNCM I-4036 on Hepatic Steatosis in Zucker Rats

**DOI:** 10.1371/journal.pone.0098401

**Published:** 2014-05-22

**Authors:** Julio Plaza-Diaz, Carolina Gomez-Llorente, Francisco Abadia-Molina, Maria Jose Saez-Lara, Laura Campaña-Martin, Sergio Muñoz-Quezada, Fernando Romero, Angel Gil, Luis Fontana

**Affiliations:** 1 Department of Biochemistry & Molecular Biology II, School of Pharmacy, University of Granada, Granada, Spain; 2 Institute of Nutrition & Food Technology “José Mataix”, Biomedical Research Center, University of Granada, Granada, Spain; 3 Department of Cell Biology, School of Sciences, University of Granada, Granada, Spain; 4 Department of Biochemistry & Molecular Biology I, School of Sciences, University of Granada, Granada, Spain; 5 Hero Global Technology Center, Hero Spain, S.A., Alcantarilla, Murcia, Spain; Catalan Institute for Water Research (ICRA), Spain

## Abstract

We have previously described the safety and immunomodulatory effects of *Lactobacillus paracasei* CNCM I-4034, *Bifidobacterium breve* CNCM I-4035 and *Lactobacillus rhamnosus* CNCM I-4036 in healthy volunteers. The scope of this work was to evaluate the effects of these probiotic strains on the hepatic steatosis of obese rats. We used the Zucker rat as a genetic model of obesity. Zucker-Lepr*^fa/fa^* rats received one of three probiotic strains, a mixture of *L. paracasei* CNCM I-4034 and *B. breve* CNCM I-4035, or a placebo for 30 days. An additional group of Zucker-lean^+/*fa*^ rats received a placebo for 30 days. No alterations in intestinal histology, in the epithelial, lamina propria, muscular layers of the ileal or colonic mucosa, or the submucosae, were observed in any of the experimental groups. Triacylglycerol content decreased in the liver of Zucker-Lepr*^fa/fa^* rats that were fed *L. rhamnosus*, *B. breve*, or the mixture of *B. breve* and *L. paracasei*. Likewise, the area corresponding to neutral lipids was significantly smaller in the liver of all four groups of Zucker-Lepr*^fa/fa^* rats that received probiotics than in rats fed the placebo. Zucker-Lepr*^fa/fa^* rats exhibited significantly greater serum LPS levels than Zucker-lean^+/*fa*^ rats upon administration of placebo for 30 days. In contrast, all four groups of obese Zucker-Lepr*^fa/fa^* rats that received LAB strains exhibited serum LPS concentrations similar to those of Zucker-lean^+/*fa*^ rats. Serum TNF-α levels decreased in the Zucker-Lepr*^fa/fa^* rats that received *B. breve*, *L. rhamnosus*, or the mixture, whereas *L. paracasei* feeding decreased IL-6 levels in the serum of Zucker-Lepr*^fa/fa^* rats. In conclusion, the probiotic strains reduced hepatic steatosis in part by lowering serum LPS, and had an anti-inflammatory effect in obese Zucker rats.

## Introduction

Obesity has reached pandemic levels and is becoming a serious health problem worldwide. In developing countries, the prevalence of obesity has tripled over the last 20 years owing to the adoption of a Western lifestyle (i.e., low physical activity levels, sedentariness, and excessive eating) [1], [2].

The increasing prevalence of type 2 diabetes (T2D), cardiovascular disease, and certain types of cancer is linked to obesity. Thus, approximately 90% of T2D cases are attributable to excess body weight, and 200 million people worldwide are estimated to have glucose intolerance and insulin resistance syndrome (IRS), a figure that is expected to rise to 420 million by the year 2025 [3].

Recent evidence indicates that the intestinal microbiota plays a crucial role in body weight and fat mass [4]–[6], and, accordingly, suggests an association between the gut microbiota and T2D [7]–[9]. Probiotics are live microorganisms that confer a health benefit on the host when administered in adequate amounts [10], although dead bacteria and bacterial molecular components may also exhibit probiotic properties. Strains belonging to *Bifidobacterium* and *Lactobacillus* are the most widely used probiotic bacteria and are included in many functional foods and dietary supplements [11]–[13]. Probiotics can modulate the gut microbiota and the mucosal immune system [14]–[16].

For probiotics to be successful, they must exhibit certain characteristics: i.e., tolerance to gastrointestinal conditions (gastric acid and bile), ability to adhere to the gastrointestinal mucosa, and competitive exclusion of pathogens [17], [18]. We have previously described the isolation of three lactic acid bacterial (LAB) strains from the feces of exclusively breast-fed newborn infants. These strains were selected based on their probiotic properties, such as adhesion to intestinal mucus, sensitivity to antibiotics and resistance to biliary salts and low pH. We identified these strains as *Lactobacillus paracasei* CNCM I-4034, *Bifidobacterium breve* CNCM I-4035, and *Lactobacillus rhamnosus* CNCM I-4036 [19].

Recently, we demonstrated the tolerance and safety of these three strains in a multi-centre, randomized, double-blind, placebo-controlled trial with healthy volunteers [20]. Oral administration of these LAB strains modified the bacterial populations in the feces of the volunteers, and all three strains exerted varying degrees of immunomodulatory effects [20]. Thus, administration of *B. breve* CNCM I-4035 resulted in a significant increase in fecal secretory IgA content. In addition, IL-4 and IL-10 was increased, whereas IL-12 was decreased, in the serum of volunteers treated with any of the three strains.

A large body of evidence has highlighted the concept that putative intestinal bacteria–derived compounds may affect liver metabolism and, therefore, cause systemic diseases [6], [21], [22]. Serum LPS levels have been proposed to increase upon obesity and steatosis, leading to a metabolic endotoxemia capable of modulating proinflammatory cytokines, as well as glucose and lipid metabolism in the liver or in the adipose tissue [23]–[26]. Endotoxemia is considered a major risk for inducing liver inflammation in nonalcoholic steatohepatitis (NASH) and nonalcoholic fatty liver disease (NAFLD) in humans [27]–[30]. NASH and NAFLD have been shown to be associated with increased gut permeability in humans [31], [32]. Cani et al. have demonstrated the alteration of gut-barrier function in genetic models of obesity [33]. Overall, these studies strongly suggest a direct link between the gut microbiota, the gut barrier, and hepatic changes.

In the present study, we used the Zucker rat as a genetic model of obesity to test the probiotic properties of our three LAB strains. We chose the Zucker rat model because it has been exhaustively characterized and exhibits symptoms of IRS that are usually found in obese humans, including hyperglycemia, glucose intolerance, hyperinsulinemia, insulin resistance, hyperlipidemia, and hepatic steatosis [34]–[38]. We focused on evaluating the effects of the LAB strains on hepatic steatosis in lean and obese Zucker rats.

## Materials and Methods

### Ethical Statement

This study was carried out in strict accordance with the recommendations in the guidelines for animal research of the University of Granada (Spain). All animals received humane care. The protocol was approved by the Committee on the Ethics of Animal Experiments of the University of Granada (Permit Number. CEEA: 2011–377).

### Microorganisms

The LAB strains *Lactobacillus paracasei* CNCM I-4034, *Bifidobacterium breve* CNCM I-4035, and *Lactobacillus rhamnosus* CNCM I-4036 have been characterized and are described elsewhere [19]. These strains were assayed for enzymatic activity and carbohydrate utilization, and they were deposited in the Collection Nationale de Cultures de Microorganismes (CNCM) of the Institute Pasteur [19].

### Experimental design

Forty-eight Zucker-Lepr*^fa/fa^* and 16 Zucker-lean^+/*fa*^ male rats weighing 168–180 g were purchased from Harlan Laboratories (Charles River, Barcelona, Spain). The rats were housed in metabolic cages with a 12-h light-dark cycle and had free access to water and food. After 5 days of adaptation, 8 Zucker-lean^+/*fa*^ and 8 Zucker-Lepr*^fa/fa^* rats were euthanized as a reference (baseline). The remaining 40 Zucker-Lepr*^fa/fa^* rats were then randomly assigned to receive 10^10^ CFUs of one of the three probiotic strains, a mixture of *Lactobacillus paracasei* CNCM I-4034 and *Bifidobacterium breve* CNCM I-4035, or a placebo by oral administration each day for 30 days. An additional group of 8 Zucker-lean^+/*fa*^ rats received placebo for 30 days. The placebo contained 67% cow's milk powder, 32.5% sucrose, and 0.56% vitamin C. The goal of this study was to examine the differences between obese rats treated with probiotics and placebo.

After the intervention, the animals were anesthetized and sedated with ketamine and xylazine. Blood was drawn from the aorta and centrifuged for 10 min at 1000×g and 4°C to separate the serum from cells. Samples of intestinal mucosa and liver were also taken.

### Intestinal histology

Ileum and colon samples were fixed with 4% paraformaldehyde for 4 h at room temperature and embedded in paraffin. Three pieces of each ileum and colon were respectively embedded in the same paraffin block. Five-µm-thick sections were obtained and routinely stained with haematoxylin-eosin for their microscopic examination. Two rats per group and 8 sections per rat were stained and examined.

### Hepatic triacylglycerol (TG) assay

Hepatic TG content was determined using a commercial kit according to the manufacturer's instructions (SpinReact, Gerona, Spain). The TG values were normalized to liver weight.

### Oil red O staining

Liver samples were fixed with 4% paraformaldehyde, cryopreserved in 30% phosphate-buffered saline (PBS)-sucrose, frozen in an isopentane liquid nitrogen bath, and embedded in OCT™ compound. Three pieces of liver from each animal were fixed and embedded in the same block. Seven µm-thick cryostat sections were obtained and stained with a solution of 0.3% Oil Red O in 60% isopropanol. Four to 8 sections per block were stained, micrographs were taken and the percentage of the micrograph area corresponding to the lipid staining was calculated using ImageJ software (National Institutes of Health, USA). Two rats per group were used for this study.

### Serum biochemistry

Concentrations of glucose, insulin, phospholipids, triacylglycerols, HDL-cholesterol, LDL-cholesterol, and non-esterified fatty acids (NEFA), as well as the activities of AST and ALT were determined in the serum of the rats using commercial kits. Relative insulin sensitivity was determined by the homeostasis model assessment of insulin resistance (HOMA-IR) as described [39].

### Serum lipopolysaccharide (LPS) concentration

Serum LPS was measured with an enzyme-linked immunosorbent assay kit from Cloud-Clone Corp., Houston, USA, following the manufacturer's directions.

### Adipokine and cytokine quantification in serum

Serum concentrations of leptin, adiponectin, TNF-α, and IL-6 were measured using MILLIplex™ immunoassays (Merck-Millipore, MA, USA) and the Luminex 200 system according to the manufacturer's instructions.

### Statistical analysis

All results are expressed as the mean ± SEM unless otherwise indicated. Statistical analyses between Zucker-Lepr*^fa/fa^* and Zucker-lean^+/*fa*^ male rats were performed using the t test at the baseline and after the intervention (placebo groups). Significant differences between obese rats that received placebo and any group of obese rats that received a specific strain after intervention were analyzed using one-factor ANOVA, which was corrected by an *a posteriori* Bonferroni test (*P*<0.05). All analyses were performed using the statistical package IBM SPSS (Statistical Package for the Social Sciences) Statistics 20 (Somers, NY).

## Results

### Zucker-Lepr*^fa/fa^* rats exhibited severe signs of insulin resistance syndrome (IRS) at the end of the intervention period

Although the body weights of Zucker-Lepr*^fa/fa^* (n = 8) and Zucker-lean^+/*fa*^ rats (n = 8) were initially similar (179.9 g±2.2 g vs. 168.9 g±4.9 g, respectively, *P*>0.3), the Zucker-Lepr*^fa/fa^* rats were clearly obese after 30 days of feeding with the placebo (Zucker-Lepr*^fa/fa^* (n = 8) 294.4 g±5.7 g vs. Zucker-lean^+/*fa*^ (n = 8) 241.5 g±5.6 g, *P*<0.001). No adverse events occurred during or after treatment.

We measured parameters related to carbohydrate and lipid metabolism, as well as hepatic function in the serum of the rats (n = 8 per group, Table 1). At baseline, the glucose, insulin, HOMA-IR, phospholipid, TG, total cholesterol, and HDL cholesterol concentrations were significantly greater in the Zucker-Lepr*^fa/fa^* rats than in the Zucker-lean^+/*fa*^ rats (*P*<0.05). With the exceptions of phospholipids and HDL cholesterol, all of these parameters were worse in obese rats that were fed the placebo for 30 days compared to the lean controls (*P*<0.05) (Table 1).

**Table 1:** Serum biochemical parameters of Zucker-lean^+/*fa*^ and Zucker-Lepr*^fa/fa^* rats fed either a placebo or LAB strains.

	Baseline		Placebo		Intervention with LAB strains			
	ZL	ZO	ZL	ZO	*L. rhamnosus*	*L. paracasei*	*B. breve*	Mixture
Glucose (mg/dL)	165.2±11.1	257.4±38.7^#^	191.1±4.9	290.1±33.2^†^	267.2±14.7	243.2±38.9	229.7±19.6	272.7±15.7
Insulin (µg/L)	0.8±0.1	3.1±1.1^#^	1.1±0.1	3.4±0.5^†^	3.8±0.6	3.9±1.0	3.5±0.4	2.6±0.4
HOMA-IR	3.1±0.5	8.0±1.1^#^	4.2±0.2	18.2±3.9^†^	13.8±2.3	9.7±2.3	13.2±1.6	11.6±2.1
Phospholipids (mg/dL)	185.4±7.6	271.9±20.8^#^	144.4±7.8	242.9±43.2^†^	283.7±14.7	239.9±49.2	318.2±16.2	323.9±23.1
Triacylglycerols (mg/dL)	54.3±4.5	152.7±23.2^#^	46.2±1.8	256.4±25.1^†^*	242.5±26.9	364±12.1	269.1±51.3	297.7±46.9
Total Cholesterol (mg/dL)	123.6±6.5	143.5±4.7^#^	99.6±3.7	174.5±13.1^†^*	191±10.9	208.8±22.8	211.3±5.4	191±10.9
HDL Cholesterol (mg/dL)	32.1±0.9	40.7±2.3^#^	18.9±2.1	23.8±7.1	39.7±5.5	28.4±8	33.7±3.4	37.3±1.8
LDL Cholesterol (mg/dL)	80.5±5.3	77.2±8.3	70.3±3.3	99.3±11.9^†^	93.9±14.2	126.6±20	108.8±7.9	111.6±12.3
NEFA (mmol/L)	0.3±0.03	0.3±0.01	0.3±0.04	0.5±0.05^†^*	0.4±0.04	0.6±0.1	0.6±0.08	0.6±0.08
AST (U/L)	110±13.5	216.1±19.9^#^	97.2±10.8	356±69.3^†^	309.7±52.6	378.7±61.6	424.3±70.9	363.6±58.5
ALT (U/L)	39.4±2.5	130.8±19.9^#^	35.8±6.1	275±52.6^†^*	235±31.4	316.8±46.3	347.9±60.3	296±61.9

Values are the means ± SEM, n = 8 per group. ^#^ *P*<0.05 (ZL baseline vs. ZO baseline), ^†^ *P*<0.05 (ZL + placebo vs. ZO + placebo), *P<0.05 (ZO baseline vs. ZO + placebo). ALT, alanine aminotransferase; AST, aspartate aminotransferase; NEFA, non-esterified fatty acids. HOMA-IR, homeostasis model assessment of insulin resistance. ZL, Zucker-lean^+/*fa*^ rats; ZO, Zucker-Lepr*^fa/fa^* rats.

The LDL cholesterol and NEFA concentrations were similar in both groups of rats at baseline but were significantly higher in Zucker-Lepr*^fa/fa^* rats that were fed the placebo for 30 days compared to Zucker-lean^+/*fa*^ rats (*P*<0.05) (Table 1). At baseline, the AST and ALT activities differed between the Zucker-Lepr*^fa/fa^* rats and the Zucker-lean^+/*fa*^ rats and remained significantly elevated in the Zucker-Lepr*^fa/fa^* rats after the intervention (t = 30) with the placebo (*P*<0.05) (Table 1). The concentrations of TG, total cholesterol and NEFA, as well as the ALT activity, of Zucker-Lepr*^fa/fa^* rats worsened after 30 days of intervention (*P*<0.05).

**Figure 1:**
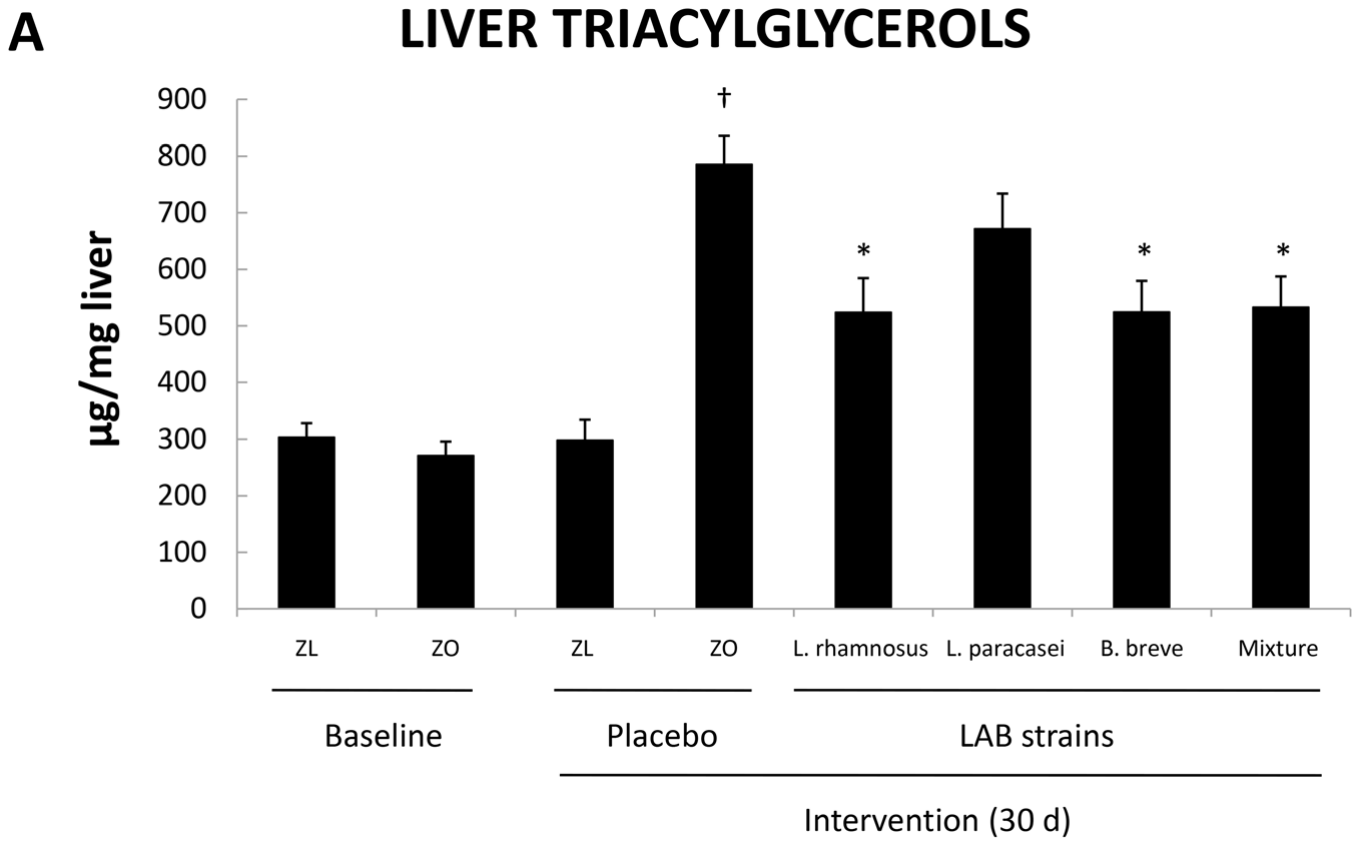
Liver triacylglycerol content of Zucker-lean^+/*fa*^ and Zucker-Lepr*^fa/fa^* rats that were fed either a placebo or LAB strains for 30 days. Values are the means ± SEM, n = 8 per group. ^†^ *P*<0.05 (ZL + placebo vs. ZO + placebo), and *P<0.05 (ZO + placebo vs. ZO + LAB strains). ZL, Zucker-lean^+/*fa*^ rats; ZO, Zucker-Lepr*^fa/fa^* rats.

The TG content was also measured in the livers of the rats (Figure 1). Hepatic TG content was similar in Zucker-Lepr*^fa/fa^* rats and Zucker-lean^+/*fa*^ rats at baseline. However, the liver TG content was 2.5-fold greater in the Zucker-Lepr*^fa/fa^* rats at the end of the intervention with the placebo (*P*<0.05) (Figure 1). Together, these results indicated that Zucker-Lepr*^fa/fa^* rats showed clear signs of IRS.

### LAB strains did not exert any effect, beneficial or detrimental, on intestinal histology

**Figure 2:**
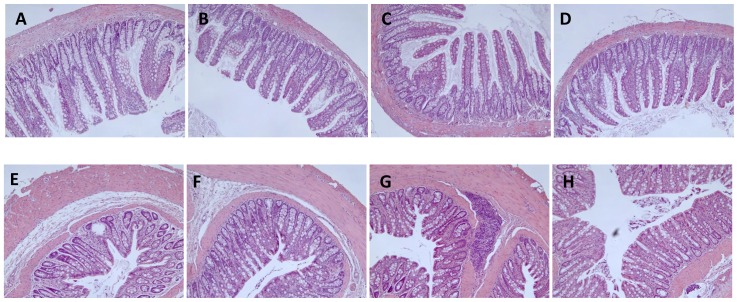
Haematoxylin-eosin stained, 5-µm-thick sections of ileal (top panels, A–D) and colonic (bottom panels, E–H) mucosa of Zucker-lean^+/*fa*^ and Zucker-Lepr*^fa/fa^* rats that were fed either a placebo or LAB strains for 30 days. Two rats per group were used for this staining. Three pieces of tissue from each animal were fixed and embedded in the same paraffin block. Four to 8 sections per block were cut, stained and analyzed. Representative micrographs from various groups are shown. A and E: Zucker-lean^+/*fa*^ rats at baseline; B and F: Zucker-Lepr*^fa/fa^* rats + placebo; C and G: Zucker-Lepr*^fa/fa^* rats +*L. rhamnosus*; and D and H: Zucker-Lepr*^fa/fa^* rats + LAB mixture.

No alterations in intestinal histology, of the epithelial, lamina propria, or muscular layers of the ileal or colonic mucosa, or in the submucosae, were observed in any of the experimental groups (Figure 2). The remaining intestinal layers also appeared normal in all of the experimental groups. These results suggested that the probiotics did not alter the morphology of this organ, reinforcing the safety of all three LAB strains.

### Steatosis was decreased in Zucker-Lepr*^fa/fa^* rats that were fed LAB strains

To investigate whether the bacterial strains affected hepatic steatosis, we measured the TG content in the liver of rats fed these strains (Figure 1). Strikingly, the TG content was significantly lower in the liver of Zucker-Lepr*^fa/fa^* rats that were fed *L. rhamnosus* CNCM I-4036, *B. breve* CNCM I-4035, or the mixture of *B. breve* CNCM I-4035 and *L. paracasei* CNCM I-4034 for 30 days (*P*<0.05) than in the liver of Zucker-Lepr*^fa/fa^* rats that were fed the placebo (Figure 1).

**Figure 3:**
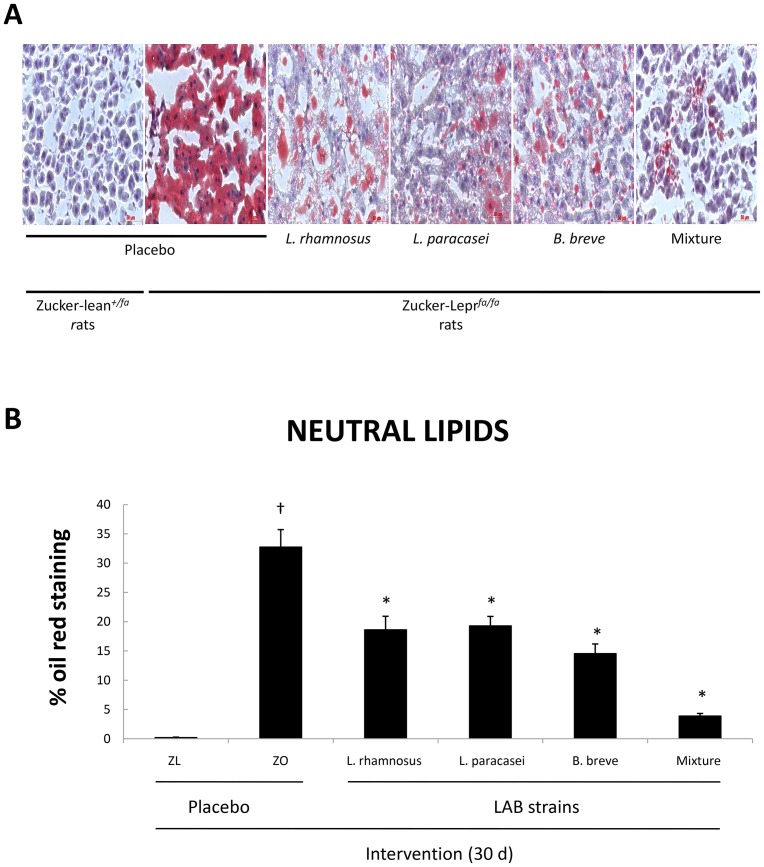
Representative micrographs of 7-µm-thick liver sections stained with 0.3% Oil red O in 60% isopropanol of Zucker-lean^+/*fa*^ and Zucker-Lepr*^fa/fa^* rats that were fed either a placebo or LAB strains for 30 days (A). Percentage of the micrograph area corresponding to the lipid staining of liver sections described in panel A was calculated (B). Values are the means ± SEM, n = 2 per group. ^†^ *P*<0.05 (ZL + placebo vs. ZO + placebo), and *P<0.05 (ZO + placebo vs. ZO + LAB strains). ZL, Zucker-lean^+/*fa*^ rats; ZO, Zucker-Lepr*^fa/fa^* rats.

These results were confirmed by Oil red O staining of liver sections (Figure 3). All four groups of Zucker-Lepr*^fa/fa^* rats that received probiotic bacteria exhibited significantly lower percentages of neutral lipids in the liver compared with Zucker-Lepr*^fa/fa^* rats fed the placebo (Figure 3).

### LAB strains did not affect serum biochemistry

No significant differences in HOMA-IR values or any of the biochemical parameters that were analyzed in the serum were found among the various groups of Zucker-Lepr*^fa/fa^* rats that received LAB strains after an intervention of 30 days (Table 1), suggesting that the bacterial strains did not affect serum markers of IRS.

### LAB strains modified the profile of serum cytokines but not serum adipokines

**Figure 4:**
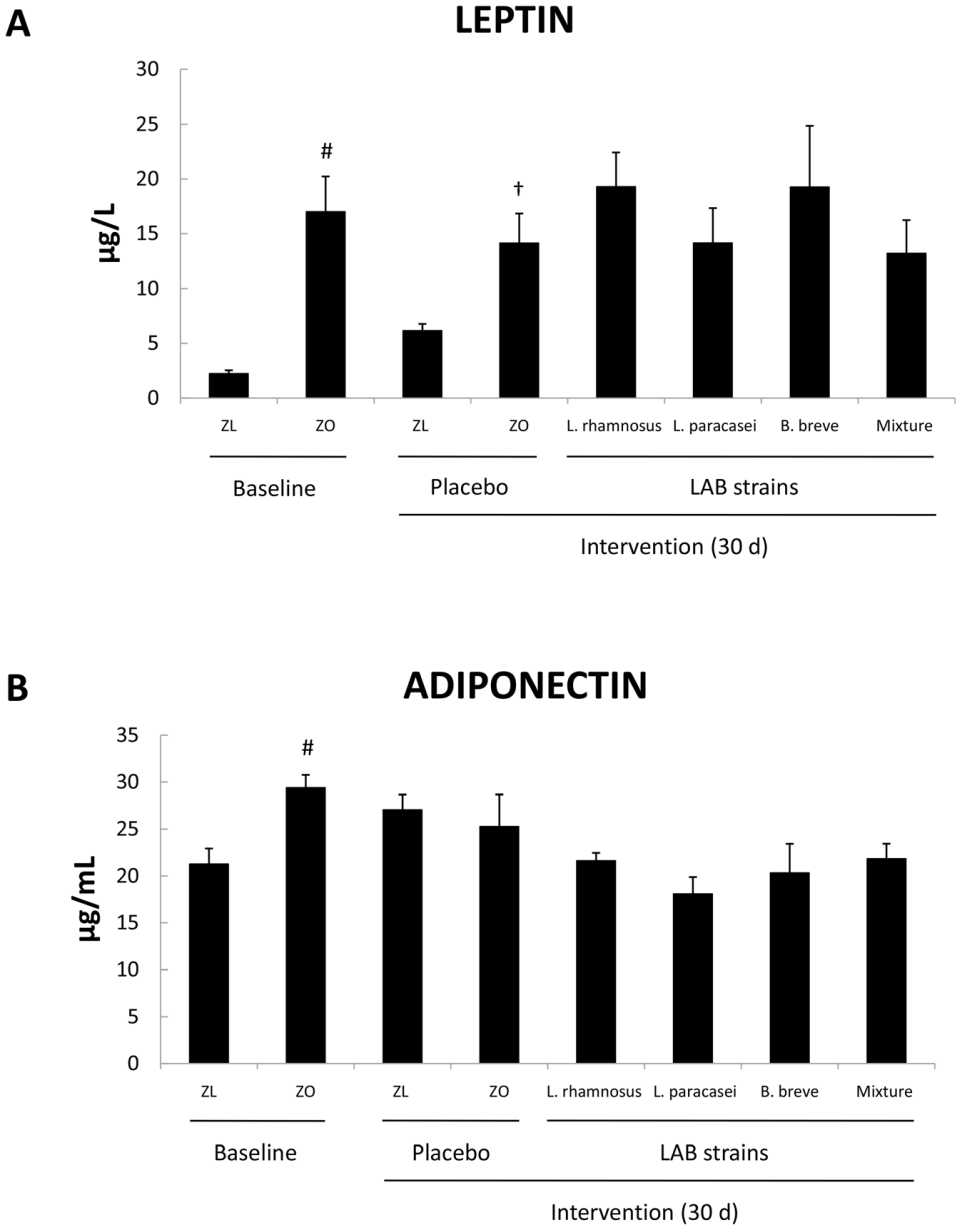
Serum leptin (A) and adiponectin (B) concentrations of Zucker-lean^+/*fa*^ and Zucker-Lepr*^fa/fa^* rats that were fed either a placebo or LAB strains for 30 days. Values are the means ± SEM, n = 8 per group. ^#^ *P*<0.05 (ZL baseline vs. ZO baseline), and ^†^ *P*<0.05 (ZL + placebo vs. ZO + placebo). ZL, Zucker-lean^+/*fa*^ rats; ZO, Zucker-Lepr*^fa/fa^* rats.

We determined the serum concentrations of leptin, adiponectin, TNF-α, and IL-6. At baseline, the concentrations of leptin and adiponectin (Figure 4) were significantly greater in Zucker-Lepr*^fa/fa^* rats than in Zucker-lean^+/*fa*^ rats (*P*<0.05). These results further supported the above serum biochemistry measurements that indicated that Zucker-Lepr*^fa/fa^* rats suffered from IRS. At the end of the intervention with the placebo, leptin concentration remained higher in the obese rats than in the lean controls (*P*<0.05) (Figure 4A). Probiotics exerted no effect on the levels of any of the adipokines that were analyzed (Figure 4).

**Figure 5:**
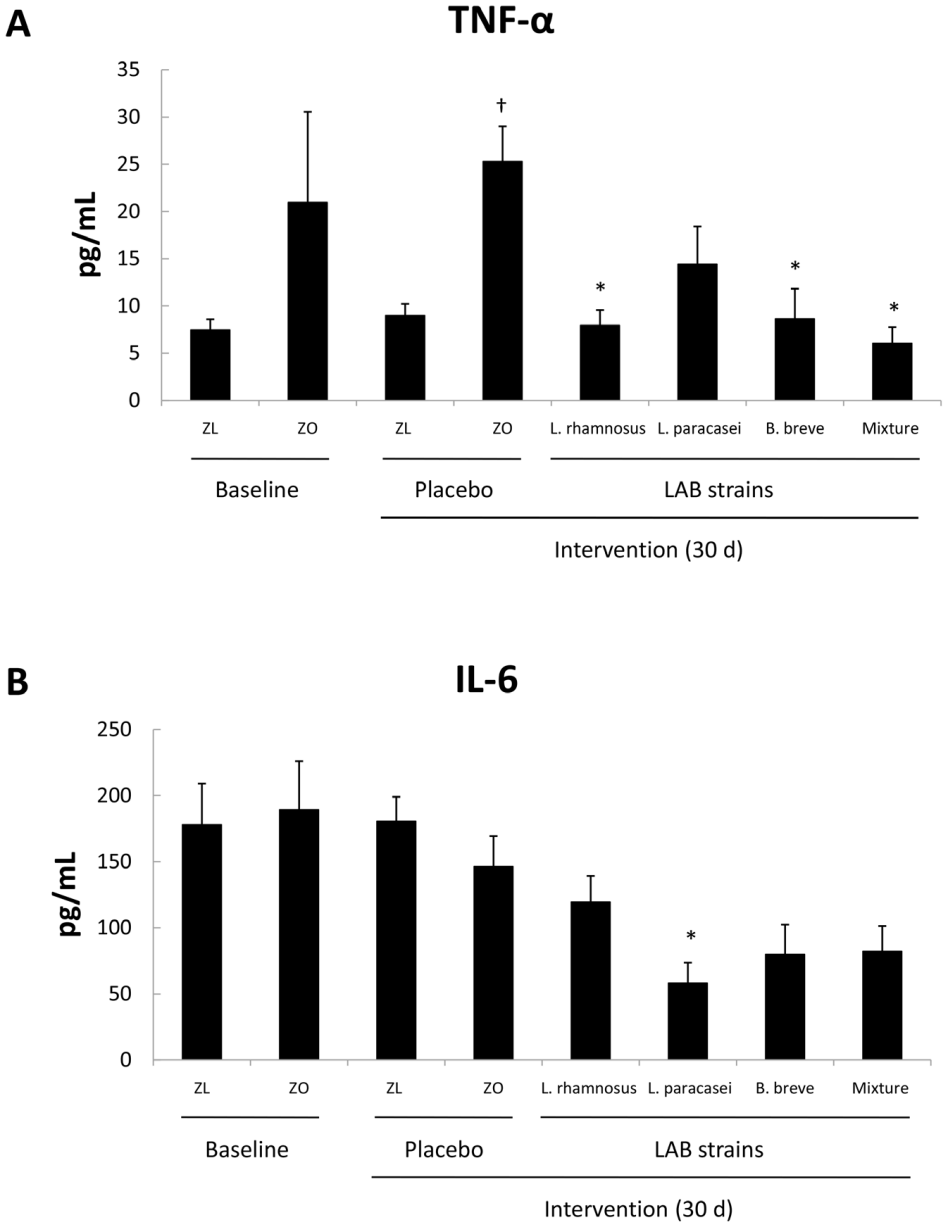
Concentrations of serum TNF-α (A) and IL-6 (B) of Zucker-lean^+/*fa*^ and Zucker-Lepr*^fa/fa^* rats that were fed either a placebo or LAB strains for 30 days. Values are the means ± SEM, n = 8 per group. ^†^ *P*<0.05 (ZL + placebo vs. ZO + placebo), and *P<0.05 (ZO + placebo vs. ZO + probiotic strains). ZL, Zucker-lean^+/*fa*^ rats; ZO, Zucker-Lepr*^fa/fa^* rats.

At baseline, the serum TNF-α and IL-6 concentrations were similar in Zucker-Lepr*^fa/fa^* and Zucker-lean^+/*fa*^ rats (Figure 5), but the TNF-α concentration was significantly increased (Figure 5A) in obese rats after 30 days of intervention with the placebo (*P*<0.05). Intervention with *L. rhamnosus* CNCM I-4036, *B. breve* CNCM I-4035, or the mixture of *L. paracasei* CNCM I-4034 and *B. breve* CNCM I-4035 decreased serum TNF-α concentrations in Zucker-Lepr*^fa/fa^* rats (*P*<0.05) (Figure 5A). The serum IL-6 levels decreased upon *L. paracasei* CNCM I-4034 administration (*P*<0.05) (Figure 5B).

### Administration of LAB strains to obese rats decreased serum LPS concentrations

**Figure 6:**
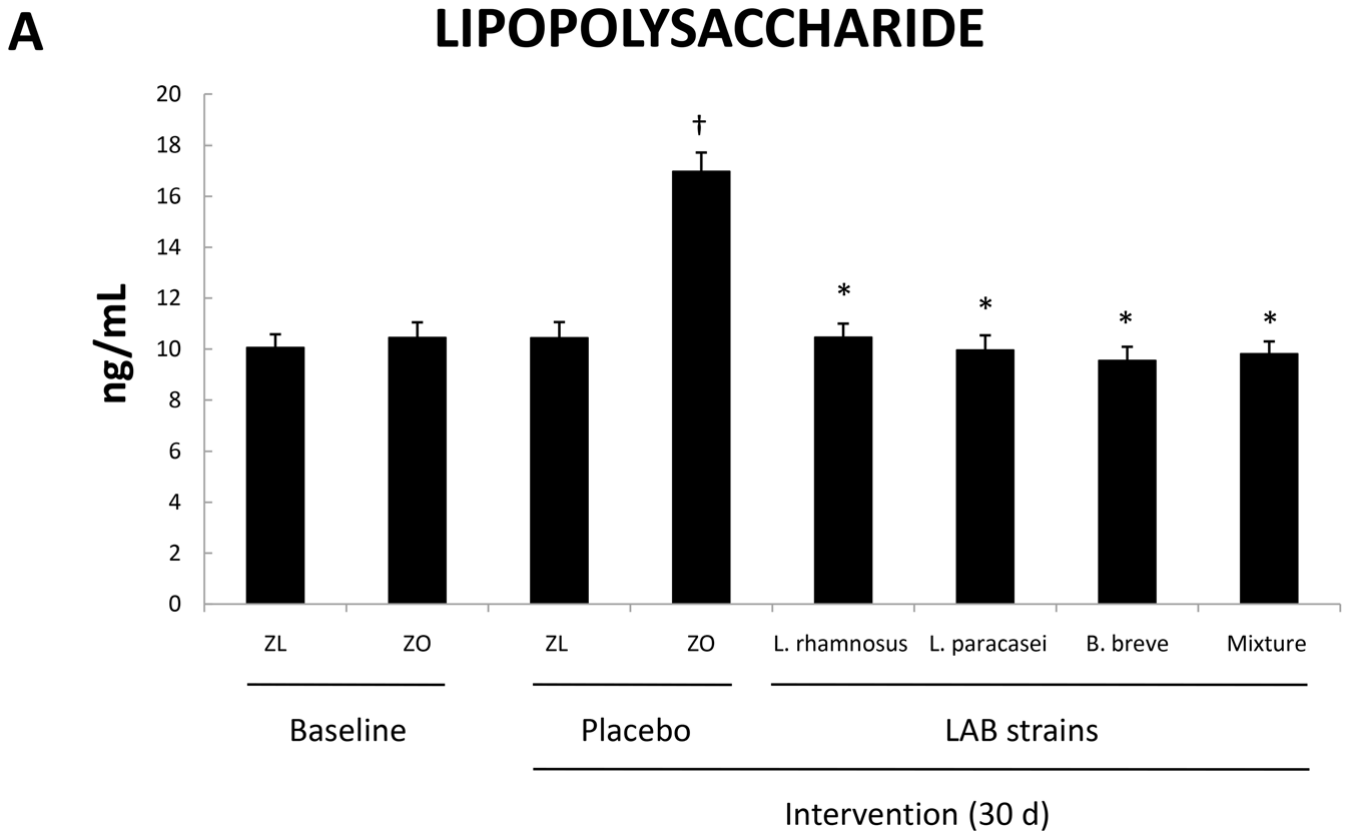
LPS concentration in serum of Zucker-lean^+/*fa*^ and Zucker-Lepr*^fa/fa^* rats that were fed either a placebo or LAB strains for 30 days. Values are the means ± SEM, n = 8 per group. ^†^ *P*<0.05 (ZL + placebo vs. ZO + placebo), and *P<0.05 (ZO + placebo vs. ZO + LAB strains). ZL, Zucker-lean^+/*fa*^ rats; ZO, Zucker-Lepr*^fa/fa^* rats.

To shed light on the potential mechanism of action of the probiotic strains we measured LPS concentrations in serum samples. These results appear in Figure 6. Zucker-Lepr*^fa/fa^* rats exhibited significantly greater LPS levels than Zucker-lean^+/*fa*^ rats upon administration of placebo for 30 days. In contrast, all four groups of obese Zucker-Lepr*^fa/fa^* rats that received LAB strains exhibited serum LPS concentrations similar to those of Zucker-lean^+/*fa*^ rats. These results matched those obtained for liver TG content (Figure 1) and clearly demonstrate the impact of probiotic administration on serum LPS.

## Discussion

Metabolic syndrome, which is better termed insulin resistance syndrome (IRS), was originally defined as concomitant hyperlipidemia, hypertension, insulin resistance and obesity [40], [41]. IRS often precedes the onset of type 2 diabetes and increases the risk of cardiovascular disease [42], [43]; therefore, IRS has become a major public health concern. The Zucker rat shows many of the features of IRS; therefore, it is one of the most commonly used genetic models of this syndrome [43]. Under our experimental conditions, Zucker-Lepr*^fa/fa^* rats exhibited obesity, hyperglycemia, insulin resistance, hypercholesterolemia, hypertriglyceridemia, and elevated serum free fatty acid concentrations after 30 days of intervention with the placebo in contrast to Zucker-lean^+/*fa*^ rats. In addition, Zucker-Lepr*^fa/fa^* rats had hepatic steatosis, as well as elevated serum AST and ALT activities, indicating that the liver component of IRS was also present in this model.

As we have previously described in human subjects [20], the administration of our three LAB strains was safe, as determined by intestinal histology, which showed no difference between LAB strain-fed rats and placebo-fed rats.

We took advantage of the Zucker rat model to investigate the effects of three probiotic strains on IRS features and inflammation. Administration of the probiotic strains did not affect serum biochemical parameters, insulin resistance, or the adipokine profile. These findings were in accordance with results recently reported in human volunteers who were fed these same three LAB strains [20].

The main finding of this study was the reduction in liver steatosis observed in obese rats fed probiotics. Zucker-Lepr*^fa/fa^* rats that received *L. rhamnosus* CNCM I-4036, *B. breve* CNCM I-4035, or a mixture of *B. breve* CNCM I-4035 and *L. paracasei* CNCM I-4034 had a liver TG content lower than rats fed the placebo. This drop in liver TG content may not be attributable to a decrease in serum insulin concentration because insulinemia was similar in Zucker-Lepr*^fa/fa^* rats fed placebo and those fed LAB strains. Additionally, given that the administration of *L. paracasei* CNCM I-4034 alone did not lower liver TG content in the obese rats, the effect observed by mixing the two probiotics might be attributable to *B. breve* CNCM I-4035. Oil red O staining of liver neutral lipids confirmed this finding in probiotic-fed obese rats.

The administration of probiotics has been reported to lower the hepatic TG and cholesterol content in mice and rats with high fat diet-induced obesity [33], [44]–[47]. To our knowledge, however, this is the first study describing the effect of probiotics on lowering the liver TG content in genetically obese Zucker rats. This anti-steatotic effect seemed to be mediated, at least in part, by the lowering of serum LPS observed in the probiotic-fed groups of obese rats. Overall our results support the current evidence that intestinal bacteria may affect liver metabolism [6], [21], [43].

Clear anti-inflammatory effects of probiotics were found in this study: i) the lower serum TNF-α concentrations found in Zucker-Lepr*^fa/fa^* rats that received *L. rhamnosus* CNCM I-4036, *B. breve* CNCM I-4035, or a mixture of *B. breve* CNCM I-4035 and *L. paracasei* CNCM I-4034 than in Zucker-Lepr*^fa/fa^* rats fed the placebo; and ii) the lower serum IL-6 concentrations in obese rats fed *L. paracasei* CNCM I-4034 than in obese rats fed the placebo. Two previous studies by our group have reported the effects of *L. paracasei* CNCM I-4034 and *B. breve* CNCM I-4035 on the production of cytokines and chemokines by cultured human intestinal dendritic cells challenged with *Salmonella typhi* [48], [49]. *L. paracasei* CNCM I-4034 decreased the amounts of proinflammatory cytokines and chemokines in these cells [48], whereas *B. breve* CNCM I-4035 was a potent inducer of pro-inflammatory factors (TNF-α, IL-8 and RANTES (Regulated on Activation, Normal T Cell Expressed and Secreted) and anti-inflammatory factors (IL-10) [49]. In the present study, the administration of *L. paracasei* CNCM I-4034 decreased the concentrations of proinflammatory cytokines such as IL-6.

Other authors have described the anti-inflammatory effects of probiotic administration. Probiotics have been reported to significantly suppress the high-fat-diet-induced activation of nuclear factor κ-B signaling that is involved in the development of high-fat-diet-induced insulin resistance [50]. In addition, the administration of lactobacilli to rats developing alcohol-induced metabolic endotoxemia and liver disease reduced plasma endotoxin levels and the liver pathology score [51]. A mixture of bifidobacteria, lactobacilli, and *Streptococcus thermophilus* has been shown to decrease liver inflammation in genetically obese mice [52] and high-fat-diet-induced hepatic inflammation in young rats [53].

Taken together, the results suggested that our probiotic strains ameliorated hepatic steatosis through a decrease in serum LPS and diminished the serum profile of proinflammatory cytokines of obese Zucker rats. These findings, along with those previously obtained by our group using these probiotic strains in *in vivo* and human studies, warrant further study to investigate the potential use of these bacterial strains as coadjuvants in the treatment of human disease.

## Supporting Information

ARRIVE Checklist S1 **ARRIVE checklist of information included in this article.**
(PDF)
